# Dr. Wu Lien Teh, plague fighter and father of the Chinese public health system

**DOI:** 10.1007/s13238-015-0238-1

**Published:** 2016-01-29

**Authors:** Zhongliang Ma, Yanli Li

**Affiliations:** School of Life Sciences, Shanghai University, Shanghai, 200444 China; Wu Lien-Teh Institute, Harbin Medical University, Heilongjiang Medical Academy, Harbin, 150081 China

**Figure 1:**
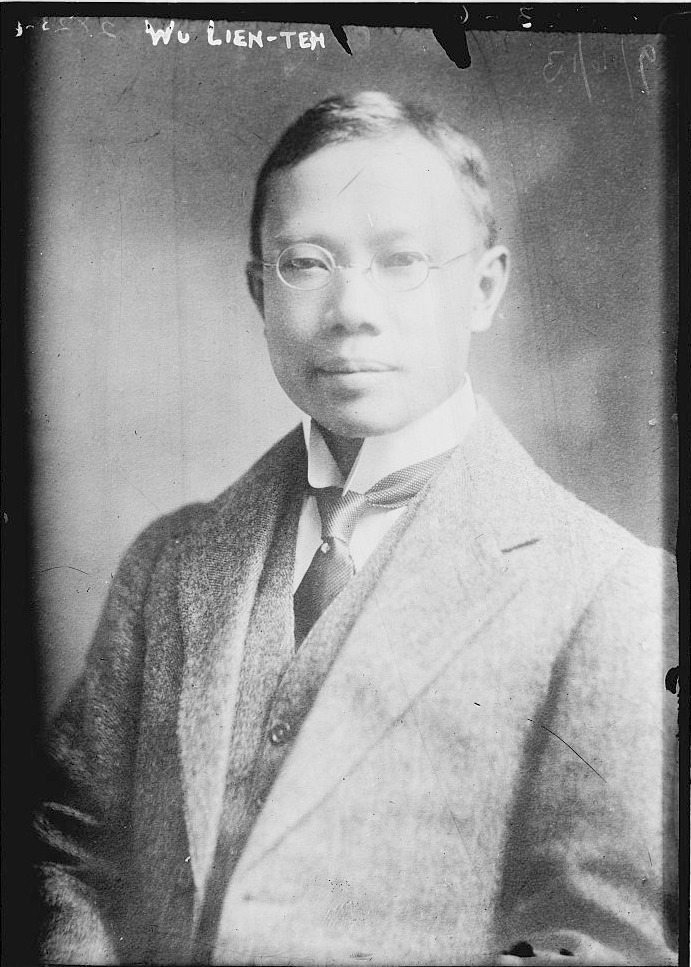
Dr. Wu Lien Teh (1879–1960)

The Chinese scholar, journalist, and philosopher, Liang Qichao said, “From late Qing Dynasty to Republic of China, about 50 years since science came into China, only Dr. Wu Lien Teh (Fig. 1) can meet and talk with foreign scientists as a real scholar.” In 1935, he also became the first Chinese doctor nominated for the Nobel Prize in Medicine.

Dr. Wu Lien Teh (Wu Liande 伍连德) was born in Penang on March 10th, 1879 and, at 17, went to England to study at Emmanuel College, at the University of Cambridge. There, he was awarded the prestigious degree of M.D. from Cambridge University.

In the fall of 1910, the deadly epidemic broke out in the northeastern region of China. The first fatality was reported in the border town of Manchouli, and the epidemic quickly spread to Harbin. Within 4 months, it had claimed over 60,000 lives.

After his arrival at Harbin, Dr. Wu performed the first-ever postmortem exam in China on a Japanese woman who had died from the epidemic. He discovered Yersinia Pestis in the body tissues and further concluded that the epidemic was pneumonic plague, which could be transmitted by human breath or sputum. This was contrary to the general idea that plague could only be transmitted by rats or fleas and could not be transmitted from person to person. His idea surprised all of his scientific peers and was met with widespread disbelief.

Dr. Mesny, a prominent French doctor, was one of those who doubted Dr. Wu’s views. Dr. Mesny himself died of pneumonic plague several days later, after refusing to wear gauze and a mask and succumbing to the epidemic infection. His death shocked the international community.

At the same time, Dr. Wu had convinced the Russian and Japanese railway authorities to cease operation of all trains in 1911. These efforts cut off all transportation, and thus transmission of the disease in Northern-East China. Nonetheless, the death toll in Harbin continued to rise as the corpses of those who had died of the epidemic served as a perfect incubator for the plague bacillus.

Dr. Wu sent a petition to sanction the cremation of the deceased, and some 3000 corpses and coffins were gathered and cremated. No further infection was reported as of March 31st, 1911. The deadly disease had vanished by the time the Chinese New Year had arrived.

**Figure 2:**
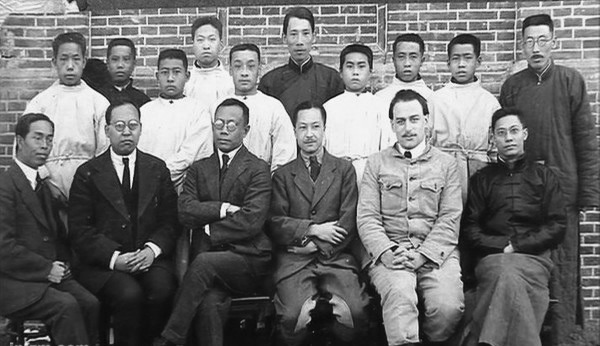
Dr. Wu Lien Teh (The 3rd from left, line 1) in The International Plague Conference (1911, in China)

On the advice of Dr. Wu, the International Plague Conference was held in Mukden from April 3rd to April 28th, 1911 (Fig. 2). Renowned epidemiologists and scientists from 11 countries, including the USA, UK, Japan, Russia, and France were in attendance. Dr. Wu was elected as president of the conference and his work on plague prevention was highly praised by all. In fact, modern medical science was established in China soon after this conference was held. Dr. Wu always stood as fighter at the forefront in the battle to prevent plague and in 1921, he successfully stamped out the recurring epidemic.

Dr. Wu devoted many of his efforts to establishing hospitals and medical colleges and founded the Chinese Medical Society. In 1931, after the Japanese invaded the northeastern provinces of China, he left for Shanghai, where he set up the National Quarantine System. He returned to Malaya with his family in 1937, following the commencement of the anti-Japanese war in China.

In his later years, after his homecoming to Malaya, he wrote and published an autobiography in English, Plague Fighter: The Autobiography of a Modern Chinese Physician. Dr. Wu passed away at the age of 81 on January 20, 1960. He was deeply mourned by the scientific and medical communities, as well as the world as a whole. The Times London commented on January 27, 1960: “By his death, the world of medicine has lost a heroic and almost legendary figure and the world at large one of whom it is far more indebted to than it knows.”

**Figure 3:**
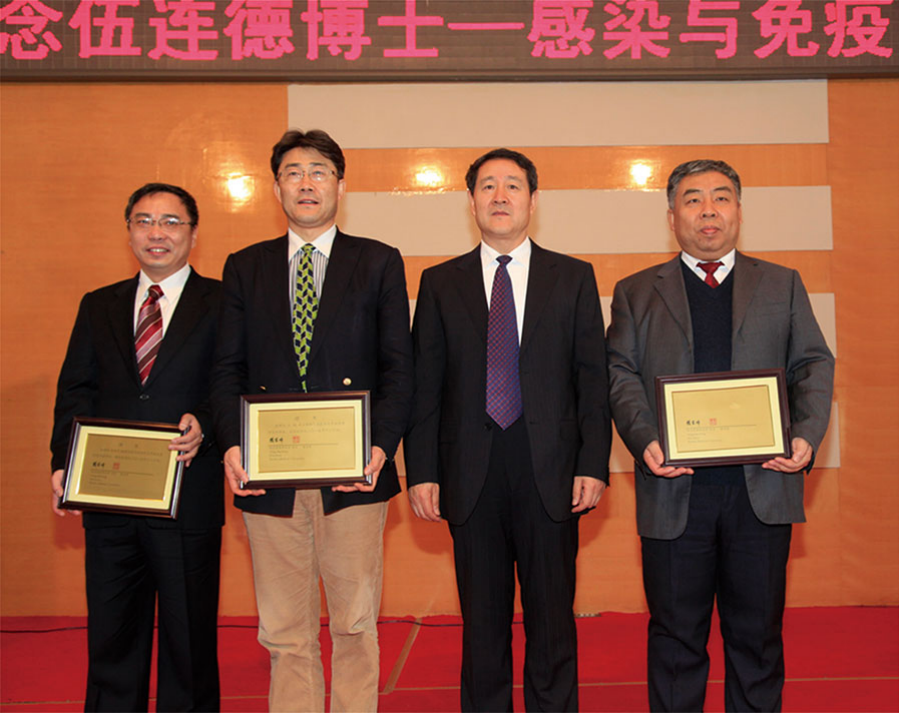
Wu Lien-Teh Institute was opened on Dec 24th, 2015 in Harbin. President of the Institute is Dr George Fu Gao (The 2nd from left)

Dr. Wu’s contributions to the development of modern medicine in China were of great importance during that era. He is regarded as the first person to modernize China’s medical services and medical education. To honor him and to remember his contributions, bronze statues of Dr. Wu Lien Teh, were erected at Harbin Medical University. He remains unforgotten and his work continues to serve us, such as occurred when SARS suddenly broke out in 2003. We owe much to Dr. Wu, who saved many lives and whose contributions to modern medicine in China allow us to continue to do so today. On Dec 24th, 2015, to thanks to his great contributions, Wu Lie-Teh Institute was opened (Fig. 3), its aims are to research on infectious diseases and share the resulting knowledge to everyone. President of Wu Lie-Teh Institute, Harbin Medical University is Dr George Fu Gao, a member of Chinese Academy of Sciences, is also a fighter to some dangours viruses, such as Ebola virus, MERS (Middle East Respiratory Syndrome) and bird flu.
